# Human Epidermal Growth Factor Receptor 2 (HER2) Expression in Colorectal Carcinoma: A Potential Area of Focus for Future Diagnostics

**DOI:** 10.7759/cureus.22811

**Published:** 2022-03-03

**Authors:** Simrandeep Kaur, Karamjit S Gill, Mridu Manjari, Surinder Kumar, Shreya Nauhria, Reetuparna Nath, Chandni Patel, Kamal Hamdan, Yujin Jeong, Narendra P Nayak, Sabyasachi Maity, Rob Hilgers, Samal Nauhria

**Affiliations:** 1 Department of Pathology, Sri Guru Ram Das Institute of Medical Sciences & Research, Amritsar, IND; 2 Department of Community Medicine, Armed Forces Medical College, Pune, IND; 3 Department of Psychology, University of Leicester, Leicester, GBR; 4 Department of Educational Services, St. George's University, St. George's, GRD; 5 Medical Student Research Institute, St. Matthew's University, Georgetown, CYM; 6 Internal Medicine, American University of Antigua, St. John's, ATG; 7 Department of Microbiology, St. Matthew's University, Georgetown, CYM; 8 Department of Physiology, St. George's University School of Medicine, St. George's, GRD; 9 Department of Pharmacology, St. Matthew's University, Georgetown, CYM; 10 Department of Pathology, St. Matthew's University, Georgetown, CYM

**Keywords:** immunohistochemistry staining, her-2/neu, trastuzumab, epidermal growth factor receptor, colorectal cancer

## Abstract

Objective

In this study, we aimed to explore the potential diagnostic utility of human epidermal growth factor receptor 2 (HER2) expression in colorectal carcinoma. We investigated the association of HER2 expression with the type and grade of the tumor along with the pattern, staining intensity, and the percentage of cells stained.

Methods

This was an observational study involving 50 cases of colorectal carcinoma that underwent immunohistochemistry to analyze the HER2 expression.

Results

The positive expression of HER2 was seen in 16 (32%) cases. The majority of the study population was between the fifth-seventh decades of life. The most commonly diagnosed tumor was conventional adenocarcinoma with grade II, cytoplasmic pattern, +2 positivity, and moderate intensity. The maximum positivity for HER2 was seen in tumors of the rectum in eight (16%) cases.

Conclusion

A substantial rate of HER2 overexpression paves the way for it to become a potential future target in cancer therapeutics.

## Introduction

Gastrointestinal (GI) cancers including esophageal, gastric, and colorectal malignancies are among the major oncological problems worldwide. Colorectal cancer (CRC) is the third most commonly diagnosed cancer worldwide, accounting for 1.9 million new cases in 2020 [1,2]. Despite advances in treatment, CRC still remains the second leading cause of cancer-related death as it is frequently associated with metastasis and recurrence [2]. In India, studies have shown a steady increase in incidence rates of CRC over recent decades, both in the younger population and older adults, raising concerns regarding an impending significant rise in CRC burden in younger adults [3].

The main treatment options currently available for CRC include surgery, chemotherapy, and radiotherapy. However, it is reported that nearly 40% of early-stage CRC patients eventually relapse after surgical resection, and the five-year survival rate of patients with advanced disease is only 10-15% [4-7]. These rising statistics of CRC regarding its high mortality rates exigently necessitate the development of novel therapeutic strategies. With recent advancements in immunology and genetic engineering, targeted receptor and immune-oncology therapies, as well as newer molecular biomarkers for rapid detection, have come to be widely discussed in the medical community [8].

The role of human epidermal growth factor receptor 2 (HER2)

Biomarkers contribute immensely to the pathologic evaluation of malignancies. These biomarkers can be used to predict how a tumor would respond to a specific therapy or to gain insight into the prognosis of the disease [9]. Significant efforts in research have resulted in the description of various putative markers, including targeted therapy for patients with CRC, namely human epidermal growth factor receptor 2 (HER2) [10,11]. HER2 pathway activation is an important mechanism of resistance for anti-epidermal growth factor receptor (EGFR) therapy, which is one of the critical therapies for various malignancies. The proto-oncogene HER2 is also known as ERBB2 or HER2/neu and is a member of the EGFR family. HER2-positive cancers arise through a pathway that is strongly associated with the amplification of the HER2 gene on chromosome 17q. HER2 is a 185-kDa transmembrane receptor tyrosine kinase that promotes cell proliferation and opposes apoptosis by stimulating the RAS- and PI3K-AKT signaling pathways [12,13]. HER2 gain-of-function mutations potentially lead to uncontrolled cell growth and division, angiogenesis stimulation, and tumor development [14].

HER2 is expressed in several tissues including epithelial cells and mammary tissue, and hence its presence is strongly implicated in breast and stomach cancers. Treating HER2-positive breast cancer patients with a monoclonal antibody (MAB), trastuzumab, has been shown to have an overall good prognosis. Recently, HER2 targeted therapy has also been increasingly used for metastatic gastric adenocarcinoma [15]. Although some studies suggest that HER2-positive CRC cases carry a poor prognosis, some clinical trials targeting the HER2 pathway have shown promising results, in which dual HER2 blockade with MABs (trastuzumab with pertuzumab) or the combination of MABs with tyrosine kinase inhibitors (trastuzumab with lapatinib) induced durable tumor response in about one-third of patients refractory to standard systemic therapy [16].

Another aspect to be noted is that HER2 expression may have a direct implication on prognosis depending on its location, either cytoplasmic or membranous. In practice, the direct target of MAB treatment in breast cancer is the membranous form of HER2, and cytoplasmic expression is irrelevant as a potential target [17]. In contrast, several studies on HER2's role in CRC have demonstrated a membranous as well as a cytoplasmic expression [18]. The definitive cause of cytoplasmic expression of HER2 still remains unclear but the upregulation of promoter-binding proteins leading to an increase in HER2 production suggests the presence of cytoplasmic expression of HER2 in CRC [19]. The fact that the latest literature supports that cytoplasmic HER2 expression in colorectal carcinoma could be associated with survival prognosis is a cause for optimism [20]. However, the definitive relationship between the prognostic and predictive value of HER2 expression and CRC is yet to be explored as HER2 overexpression has shown a wide range of variability (between 0-84%) in various CRC studies [18].

This variability may be attributed to the lack of a universally acknowledged, standardized protocol in reporting HER2 expression in CRC, resulting in studies using different antibodies, different scoring systems for the interpretation of results, or having different sample sizes [8]. A significant factor is that the variation of CRC molecular signature, as almost all cancer types, largely depends on the patient’s genomic make-up and the individual microenvironment (i.e., lifestyle and diet). Therefore, it is important to correlate the HER2 biomarker in terms of prognosis and/or predictive value with the unique characteristics of the patients' particular geographical locations worldwide, e.g., India [17].

Scarce data is available with respect to the expression of HER2 in patients with CRC in the literature, particularly from the Indian subcontinent. Therefore, in this study, we attempted to investigate the expression of HER2 in CRC in the Indian population.

## Materials and methods

Ethical approval and the recruitment of participants

The study involved 50 histopathological proven cases of CRC diagnosed in the Department of Pathology, Sri Guru Ram Das Institute of Medical Sciences & Research, Amritsar, India. The study was approved by the Institutional Ethics Committee (IEC) of the institute, and informed consent was obtained from all participants.

Managing the tissue and preparing the microscope

The colectomy specimens were placed in 10% neutral buffered formalin and transferred to the histology section. The paraffin-embedded tissue blocks were retrieved from the archives. Relevant clinical data of the patients were recorded as per the approved proforma. Further, sections were cut and stained with hematoxylin and eosin stain and studied under the light microscope for classification and histopathological grading. Sections of tissue were cut at a 3-5-μm thickness and placed on poly-l-lysine-coated slides. Immunohistochemistry of the tumors was performed for HER2 antibody as described below. As per the World Health Organization (WHO) guidelines, the histopathological grading of the tumors was divided into grade I (well-differentiated), grade II (moderately differentiated), and grade III (poorly differentiated).

HER2 expression in CRC was investigated using the conventional immunohistochemistry protocol. The tissues were deparaffinized, rehydrated, and antigen retrieval was performed. In the moisturization chamber, the sections were incubated with the mouse primary MAB against HER2 (c-erbB-2 oncoprotein, clone SP3; Diagnostic Biosystems, Pleasanton, CA) for one hour. Furthermore, the sections were placed in secondary antibodies with solution A (amplifier) and solution B as polymer. The staining was controlled using HER2-positive breast cancer tissue.

Two trained pathologists independently evaluated the HER2-stained slides based on the American Joint Committee on Cancer Staging (stage I-IV) guidelines. The following three criteria were adhered to:

1. The intensity of staining: weak/moderate/strong

2. Percentage of cells stained: 10-40% cells stained as score 1+; 40-70% cells stained as score 2+; and >70% cells stained as score 3+

3. Pattern of HER2 expression: membranous/cytoplasmic/membranous + cytoplasmic

Statistical analysis

The statistical analysis was performed using SPSS Statistics version 21 (IBM, Armonk, NY). The data were recorded for various variables such as patient age and gender, histopathological type, grade, HER2 expression, and stage of the tumor. The chi-squared test was used to investigate the correlation of HER2 in CRC. A p-value <0.05 was considered statistically significant.

## Results

Table 1 shows the demographic data of the included patients. In the present study, there was a slight preponderance of males over females with 26 (52%) males and 24 (48%) females with the M:F ratio being 1.08:1. The subjects were aged between 30-80 years. The highest incidence was observed in patients in their fifth to seventh decades of life.

**Table 1:** Demographic data of the included cases (n=50) HER2: human epidermal growth factor receptor 2

Characteristics			Number of patients	Percentage (%)
Age (years)	31-40		11	22
	41-50		6	12
	51-60		18	36
	61-70		12	24
	71-80		3	6
Gender	Male		26	52
	Female		24	48
Symptoms at presentation	Abdominal pain, constipation		2	4
	Abdominal pain		15	30
	Bleeding per rectum		19	38
	Abdominal pain, bleeding per rectum		6	12
	Constipation		6	12
	Constipation, bleeding per rectum		1	2
	Bleeding per rectum, diarrhea		1	2
Site of lesion	Caecum		3	6
	Ascending colon		9	18
	Transverse colon		5	10
	Sigmoid		14	28
	Rectosigmoid		6	12
	Rectum		13	26
Size of tumor (cm)	<5		16	59.2
	6-10		10	37
	11-15		1	3.7
Invasion	Depth of invasion	Serosa involved	16	59.26
		Muscle involved	11	40.74
	Vascular invasion	Present	20	74
		Absent	7	25.9
	Perineural invasion	Present	1	3.7
		Absent	26	96.3
Lymph node status	Metastatic		9	33.3
	Reactive		18	66.7
Histological type	Conventional adenocarcinoma		38	76
	Mucinous adenocarcinoma		10	20
	Signet ring cell carcinoma		2	4
Histological grade	Grade I (well-differentiated)		4	10.5
	Grade II (moderately differentiated)		29	76.3
	Grade III (poorly differentiated)		5	13.1
HER2 immunohistochemistry	Present		167	32
	Absent		34	68

HER2 immunohistochemistry

**Figure 1:**
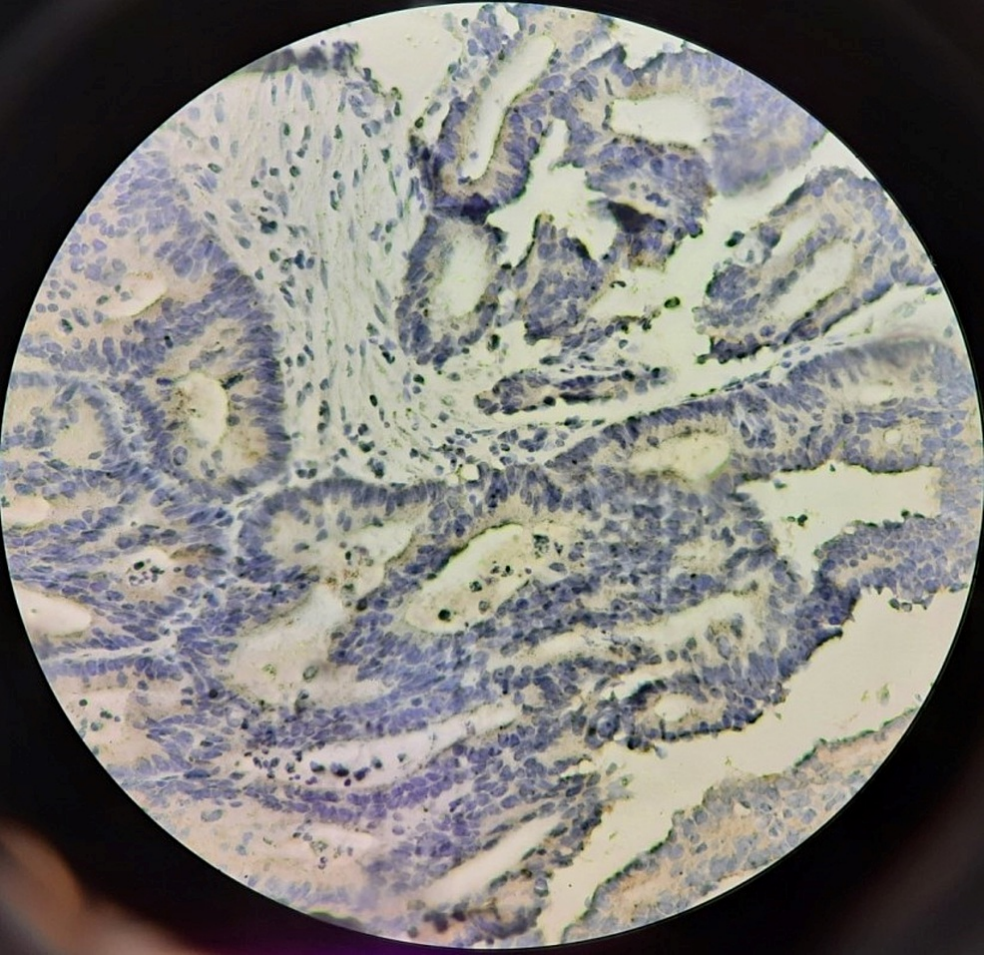
Picture micrograph showing the cytoplasmic pattern on HER2 staining with moderate intensity (IHC; 400X) HER2: human epidermal growth factor receptor 2

**Figure 2:**
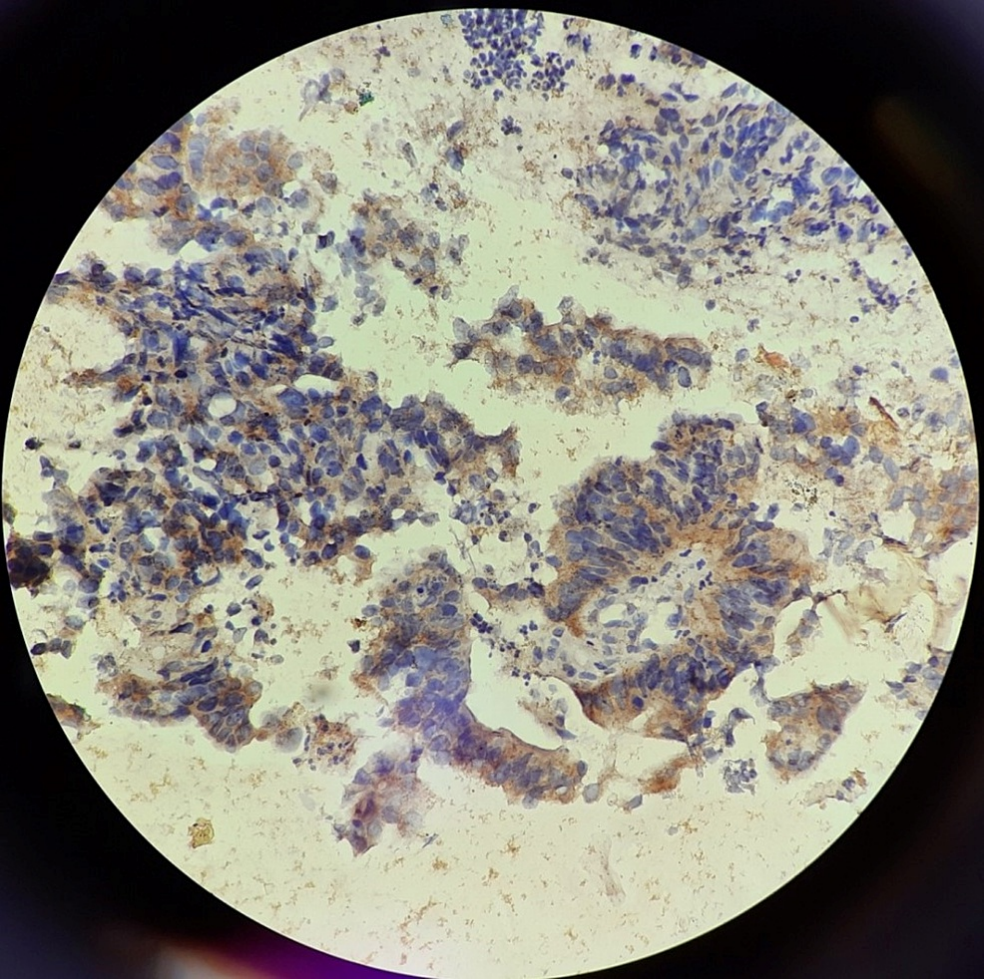
Picture micrograph showing the cytoplasmic pattern of HER2 staining with strong intensity (IHC; 400X) HER2: human epidermal growth factor receptor 2

In this study, 16 cases (32%) were positive and 34 cases (68%) were negative for HER2 staining. The pattern of staining was found to be cytoplasmic in the majority of cases with only one case of mucinous adenocarcinoma, which showed a membranous + cytoplasmic pattern. Table 2 shows the patterns of staining in different histologic types of CRC. Figure 1 and Figure 2 show a cytoplasmic expression of HER2 with moderate and strong intensity, respectively.

**Table 2:** Pattern of HER2 staining in different types of tumors HER2: human epidermal growth factor receptor 2

Type of tumor	Total patients		The pattern of HER2 staining							
			Negative		Cytoplasmic		Membranous + cytoplasmic		Membranous	
	N	%	N	%	N	%	N	%	N	%
Conventional adenocarcinoma	38	76	24	48	14	28	0	0	0	0
Mucinous adenocarcinoma	10	20	9	18	0	0	1	2	0	0
Signet ring cell carcinoma	2	4	1	2	1	2	0	0	0	0
Total	50	100	34	68	15	30	1	2	0	0

**Figure 3:**
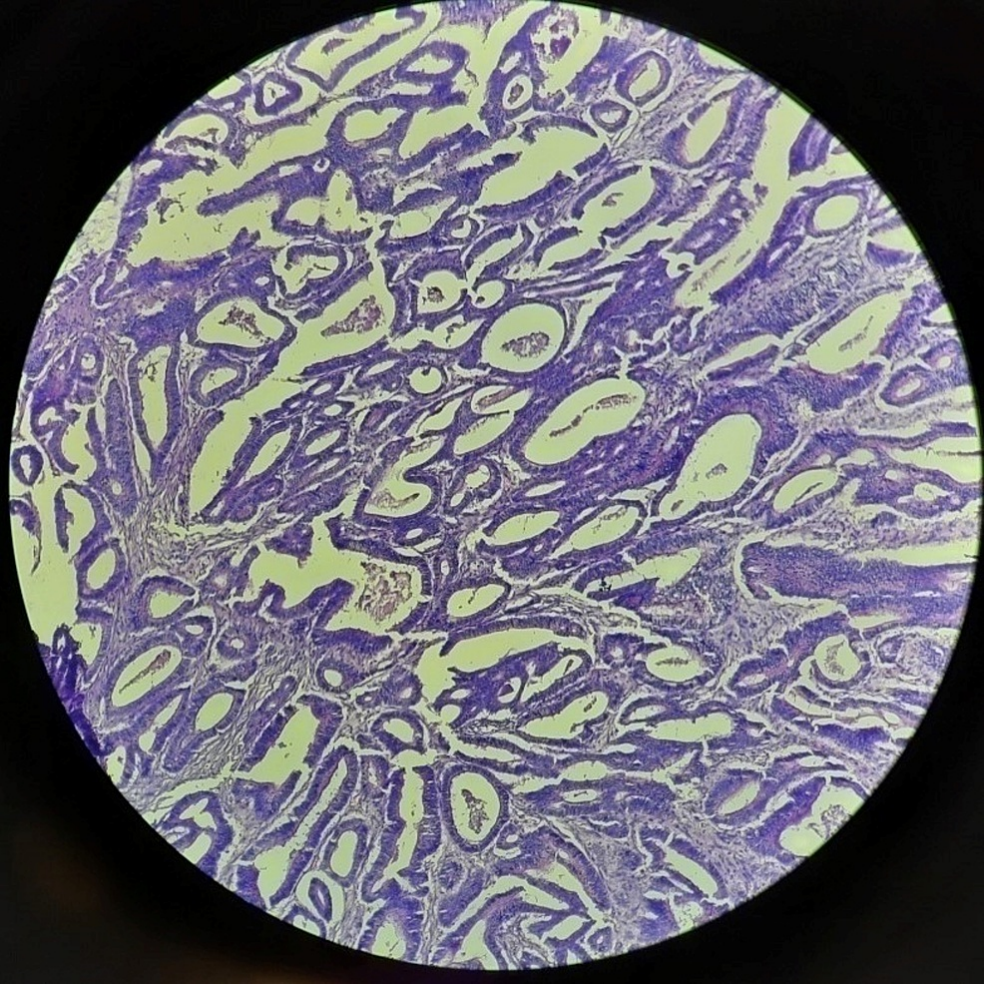
Picture micrograph showing conventional adenocarcinoma (H&E; 100X)

Out of the 38 cases of conventional adenocarcinoma, seven cases were scored as +2 and five cases were scored as +3. Out of 10 cases of mucinous adenocarcinomas, only one case was scored as +1. One out of two cases of signet ring cell carcinoma was scored as +2. Table 3 shows the percentage of cells showing staining in different types of CRC. Figure 3 shows a microscopic picture of a conventional adenocarcinoma.

**Table 3:** Percentage of cells showing staining in different types of tumors

Type of tumor	Total patients		Percentage of cell staining							
			Negative		+1 (10-40%)		+2 (40-70%)		+3 (>70%)	
	N	%	N	%	N	%	N	%	N	%
Conventional adenocarcinoma	38	76	24	48	2	4	7	14	5	10
Mucinous adenocarcinoma	10	20	9	18	1	2	0	0	0	0
Signet ring cell carcinoma	2	4	1	2	0	0	1	2	0	0
Total	50	100	34	68	3	6	8	16	5	10

Among the 38 (76%) cases of conventional adenocarcinoma, 10 cases showed moderate intensity; one case out of 10 cases of mucinous adenocarcinoma showed moderate intensity and only one case out of two cases of signet ring cell carcinoma showed moderate intensity. Out of four cases with grade I, one case showed +1 positivity and one case showed +2 positivity. Out of 30 cases with grade II, two cases showed +1 positivity, six cases showed +2 positivity, and five cases showed +3 positivity. None of the five cases with grade III showed positivity for HER2. Table 4 shows the percentages of HER2 staining in different grades of tumors.

**Table 4:** Percentage of HER2 staining in different grades of tumors HER2: human epidermal growth factor receptor 2

Percentage of cell staining	Percentage of HER2 staining						Total	
	Grade I		Grade II		Grade III			
	N	%	N	%	N	%	N	%
Negative	2	5.1	17	43.6	5	12.8	24	61.5
+1 (10-40%)	1	2.5	2	5.1	0	0	3	7.6
+2 (41-70%)	1	2.5	6	15.3	0	0	7	17.9
+3 (>70%)	0	0	5	12.8	0	0	5	12.8
Total	4	10.3	30	76.9	5	12.8	39	100

We also correlated the HER2 expression with age, gender, and tumor size, and the results are shown in Table 5.

**Table 5:** Correlation of HER2 status with age, gender, and tumor size HER2: human epidermal growth factor receptor 2

Demographics	Parameters	Total number of cases		IHC staining								P-value (chi-squared)
		N	%	Negative		+1 (10-40%)		+2 (40-70%)		+3 (>70%)		
				N	%	N	%	N	%	N	%	
Age group (years)	31-40	11	22	8	16	1	2	0	0	2	4	0.152
	41-50	6	12	3	6	1	2	1	2	1	2	
	51-60	18	36	15	30	1	2	1	2	1	2	
	61-70	12	24	7	14	0	0	5	10	0	0	
	71-80	3	6	2	4	0	0	0	0	1	2	
Gender	Male	26	52	17	34	3	6	5	10	1	2	0.216
	Female	24	48	18	36	0	0	3	6	3	6	
Tumor size (cm)	<5	16	59.3	14	51.8	0	0	2	7.4	0	0	0.564
	6-10	10	37	8	29.6	1	3.7	0	0	1	3.7	
	11-15	1	3.7	1	3.7	0	0	0	0	0	0	

On correlating HER2 with site and location of the tumors, it was observed that the maximum positivity for HER2 was seen in tumors of the rectum in eight cases (16%), followed by five (10%) cases involving ascending colon and four (8%) cases involving the sigmoid colon. In all the 27 resected cases, the depth of invasion of tumor cells and perineural and vascular invasion of tumor cells were looked for and reported accordingly, and the correlation with HER2 expression was checked. It was observed that none of these three parameters had any significant correlation with HER2 expression as indicated by their p-values, which were 0.332, 0.277, and 0.718 respectively (not significant).

One out of nine cases showing metastasis in lymph nodes was positive for HER2 while two out of 18 cases showing reactive changes were positive for HER2. There was no significant correlation between lymph node status and HER2 expression (p=1.00, not significant).

## Discussion

This study focused on investigating and analyzing HER2 expression in CRC in relation to various clinicopathological variables in the Indian population. CRC is one of the most prevalent cancers worldwide among both men and women. There are many key factors that contribute to the risk of developing CRC. One of the major factors is genetic, and the most common syndromes linked with CRC are Lynch syndrome and familial adenomatous polyposis. CRC usually present with bleeding per rectum, changes in bowel movements such as diarrhea, constipation, persistent abdominal discomfort, and the feeling that one’s bowel is not completely empty, as well as unexplained weight loss. Diagnosing CRC by light microscopy has limitations in terms of predicting the prognosis.

In this study, we looked at the expression of HER2, which is a proto-oncogene located on chromosome 17 and encodes ErbB-2. It is activated by gene amplification in human cancer, where a small fragment on chromosome band 17q12-q21 can be multiplied in a cell by up to 50-100 folds. What is noteworthy in our study is that out of the total 50 cases, 16 (32%) cases stained positive for HER2 and 34 (68%) cases had negative staining. Of the 16 positive cases, the pattern of staining was found to be cytoplasmic in the majority of cases (93%) while one case showed a membranous + cytoplasmic pattern. A similar finding was reported by a previous study, which observed a majority (48%) of CRCs with a cytoplasmic expression of HER2 and a relatively smaller number (26.6%) of membranous HER2-positive expression [8]. Indeed, it has been previously acknowledged that HER2 positivity in CRC is highly variable with similar high variability in expression patterns. Factors that contribute to this reported data inconsistency include a lack of globally standardized protocol for reporting HER2 expression in CRC as well as discrepancies among studies conducted in different geographical regions of the world with different confounding factors such as lifestyle and diet [21].

Gathering extensive data with regard to HER2-related CRC is crucial due to its potential therapeutic implications. It is well known that current MAB therapy for HER2-related breast cancer distinguishes the histopathologic differences between the membranous and cytoplasmic expression of HER2, the latter being clinically irrelevant as a potential target [8]. While there have been a handful of promising results indicating the clinical potential of therapeutic HER2 blockade, the connection between the cellular location of HER2 expression and the success of HER2-targeting agents against CRC is yet to be established. Future studies on the aforementioned relationship may reveal exciting new possibilities of effective treatments specifically tailored to each patient with different types of HER2-positive CRC.

In the present study, we examined the correlation of HER2 status with gender, age, and tumor size. Our cohort had 26 (52%) males and 24 (48%) females, which was similar to a previous study [22]. In the same study, researchers noticed that as the patient’s age increased, the percentage of HER2 staining also increased, which was statistically significant. Another study has agreed with our findings, where they found no statistically significant correlation between HER2 expression and age [8]. Previous research has reported a slight male preponderance with a majority of cases being in the fifth to seventh decades of their lives, which aligns with our findings [22-24]. However, another study reported a high number of females in the included patient sample with a mean age of 71 years [25]. In a similar vein, another study did not find statistical significance when examining the correlation of HER2 with age and gender.

In the present study, 38 (76%) cases were diagnosed to be conventional adenocarcinoma, 10 (20%) cases were mucinous adenocarcinoma, and the remaining two (4%) cases were diagnosed as signet ring cell carcinoma. Similar findings were observed in another study where most of the cases were conventional adenocarcinoma (77.5%) followed by mucinous adenocarcinoma (17.5%) and carcinoid and signet cell carcinoma [22]. In comparison to this, another study observed that 13 (13.7%) tumors were mucinous while 82 (86.3%) were non-mucinous variants [8].

Bleeding per rectum and abdominal pain were the chief complaints in the present study. Out of 50 cases, 19 (38%) presented with bleeding per rectum and 15 (30%) with abdominal pain, while the rest of the cases had multiple complaints in combination with pain in the abdomen. Similar findings were observed in a study where a majority (95%) of the patients presented with bleeding per rectum with pain or burning sensation during defecation [24]. Previous studies have reported that the descending colon was the most common site involved in terms of tumor location, whereas in the present study, the sigmoid colon (n=14, 28%) and rectum (n=13, 26%) were the most common sites involved, followed by ascending colon in nine (18%) cases [22,23]. Among the 27 hemicolectomy specimens in the present study, a majority (16) of the cases had tumors <5 cm in size, 10 cases had tumors 6-10 cm in size, and one case had a tumor 11-15 cm in size. Similar findings were reported by a study in which the majority of the cases had a tumor size of 3 cm [23]. In our study, out of the 27 (54%) resected specimens, the lymph node status showed only nine (18%) cases with metastatic deposits while 18 (36%) were reactive. One study found 19 (38%) cases involved with metastatic nodal deposits while 13 (26%) had distant metastasis [23]. In another study, the proportion of metastatic cases was high (50%) while another 50% were reactive [22].

A previous study has reported HER2 positivity in 22% of patients and a significant correlation between HER2 expression and advanced cancer stage [26]. The same study found a statistically significant correlation between HER2 overexpression and the tumor size as well as tumor grade. In our study, we correlated various grades of the tumor with HER2 expression but did not find any significant association. Contrary to our findings, previous studies have observed increased HER2 positivity with an increase in the grade of the tumor [22,24]. Although HER2 expression decreased with an increase in grade in the present study, which is different from other studies, since HER2 positivity indicates a poor prognosis, patients can be treated accordingly.

The results of our study also indicate that there is no correlation between the site of the tumor and HER2 expression, which is in accordance with previous studies demonstrating no significant association between HER2 expression and tumor location [27,28]. Evidence from previous literature suggests that future investigations may shift focus to other factors affecting HER2 positive CRC rather than classifying patient groups based on tumor location.

There are a few limitations to the current study. Firstly, similar to previous studies mentioned above, the sample size was small, which jeopardizes the results due to sampling errors. The results from the chosen sample of patients may not be representative of the broader Indian population. Secondly, a large discrepancy between sample sizes of different grades of a tumor may also contribute to contradictory findings. To develop a full picture of the relationship between various tumor characteristics and HER2 positivity, additional investigations are strongly recommended to obtain more viable data for a worldwide meta-analysis.

## Conclusions

The current study was conducted to gain insights into the HER2 expression on CRC. A low rate of HER2 expression indicates the need for more standardized studies to understand the biological behavior of HER2-positive CRC. There is a need for further studies involving larger sample sizes in various geographical regions of the Indian subcontinent to draw more conclusive evidence of HER2 expression in CRC. This can potentially lead to the application of therapies involving trastuzumab and/or small inhibitors of ErbB-2 in HER2-positive CRC patients.
